# Region-Specific Roles of TGF-β2 and Angiotensin II in Fibrotic and Inflammatory Remodeling of the Optic Nerve Head

**DOI:** 10.3390/cells14221830

**Published:** 2025-11-20

**Authors:** Si-Eun Oh, Jie-Hyun Kim, Se-Eun Park, Chan-Kee Park, Hae-Young Lopilly Park

**Affiliations:** 1 Department of Ophthalmology, Bucheon St. Mary’s Hospital, College of Medicine, The Catholic University of Korea, Bucheon 14647, Republic of Korea; 2 Department of Ophthalmology, Seoul St. Mary’s Hospital, College of Medicine, The Catholic University of Korea, Seoul 06591, Republic of Korea

**Keywords:** glaucoma, optic nerve head, TGF-β2, angiotensin II, extracellular matrix remodeling, neuroinflammation

## Abstract

This study investigated the region-specific roles of transforming growth factor-β2 (TGF-β2) and angiotensin II (AngII) in extracellular matrix (ECM) remodeling and inflammatory responses within scleral tissues surrounding the optic nerve head (ONH), using primary human fibroblasts from posterior sclera, peripapillary sclera (ppScl), and fibroblast-like cells from lamina cribrosa (LC). In vivo validation was performed in a chronic ocular hypertension rat model. Fibrotic and inflammatory markers were analyzed by Western blotting, quantitative PCR, and immunocytochemistry following TGF-β2 or AngII stimulation, and in vivo effects were assessed after subtenon injection of pathway-specific inhibitors. TGF-β2 induced robust upregulation of α-smooth muscle actin, collagen type I, and fibronectin across all scleral regions, whereas AngII elicited regionally confined pro-inflammatory responses, particularly in the LC and ppScl, characterized by increased cyclooxygenase-2 expression. Inhibition of either pathway reduced ECM deposition in vivo, but only AngII blockade significantly attenuated glial activation and preserved retinal ganglion cells. These findings demonstrate that TGF-β2 predominantly drives fibrosis, while AngII promotes region-specific neuroinflammation, and that inflammation, rather than fibrosis alone, plays a critical role in glaucomatous neurodegeneration. Targeting both fibrotic and inflammatory mechanisms in a region-specific manner may offer improved neuroprotection in glaucoma.

## 1. Introduction

Glaucoma is a leading cause of irreversible blindness worldwide, characterized by the progressive loss of retinal ganglion cells (RGCs) and degeneration of the optic nerve [1,2]. While elevated intraocular pressure (IOP) remains the most important modifiable risk factor, the pathogenesis of glaucoma is multifactorial and involves a complex network of interrelated mechanisms [3]. These include impaired ocular perfusion, alterations in extracellular matrix (ECM) remodeling and neuroinflammation. Rather than acting independently, these factors often interact and potentiate one another, contributing to structural and functional damage of the optic nerve head (ONH) [4,5,6,7].

Transforming growth factor-β (TGF-β) isoforms (TGF-β1, -β2, and -β3) share receptors and signaling pathways and all contribute to fibrosis. Among them, TGF-β2 is the predominant isoform in ocular tissues and is consistently elevated in the aqueous humor and ONH of glaucomatous eyes [8]. TGF-β2 serves as a central mediator of pathological extracellular matrix (ECM) remodeling in the eye [9]. In glaucoma, TGF-β2 drives ECM deposition in both the trabecular meshwork and the ONH. Within the trabecular meshwork, this remodeling increases resistance to aqueous outflow, thereby contributing to elevated IOP [10]. In the ONH, elevated TGF-β2 is associated with reactive astrocytes, contributing to fibrotic changes that may alter biomechanical properties of the sclera and lamina cribrosa (LC). These changes can increase the susceptibility of the ONH to IOP-induced deformation and subsequent axonal damage [10,11]. Elevated IOP itself can induce ECM remodeling in both the LC and peripapillary sclera (ppScl), as demonstrated in primate models [12]. This process involves mechanosensitive responses mediated by signaling molecules such as TGF-β and matrix metalloproteinases (MMPs), which are upregulated in glaucomatous tissues and contribute to pressure-induced fibrotic and degradative changes in the ONH microenvironment [2,8,13].

Angiotensin II (AngII) is a peptide hormone of the renin-angiotensin system (RAS) that plays an important role in regulating blood pressure and fluid balance [14]. The role of AngII in the eye has gained increasing attention following the discovery of a local RAS within ocular tissues [15]. Elevated AngII expression activates retinal cells and scleral fibroblasts, implicating it in the development of ocular inflammation and fibrosis [16]. We previously demonstrated that systemic hypotension could induce AngII upregulation in the sclera, leading to RGC loss through a process involving scleral fibrosis. AngII contributes to this pathology by promoting ECM remodeling, which alters the biomechanical properties of the sclera and increases its susceptibility to glaucomatous damage [17].

In this study, we aimed to elucidate the fibrotic and inflammatory responses of human scleral fibroblasts from distinct anatomical regions—the posterior sclera, ppScl, and the LC—under stimulation with TGF-β2, AngII, and mechanical pressure. Furthermore, we evaluated the in vivo efficacy of TGF-β2 and AngII pathway inhibition in an experimental rat model of ocular hypertension. By characterizing regional differences in fibroblast behavior and testing targeted inhibition strategies, this study seeks to expand our understanding of scleral remodeling in glaucoma pathophysiology and identify potential antifibrotic and anti-inflammatory therapeutic targets.

## 2. Materials and Methods

### 2.1. Isolation of Human Scleral Fibroblasts and Lamina Cribrosa Cells

**Figure 1:**
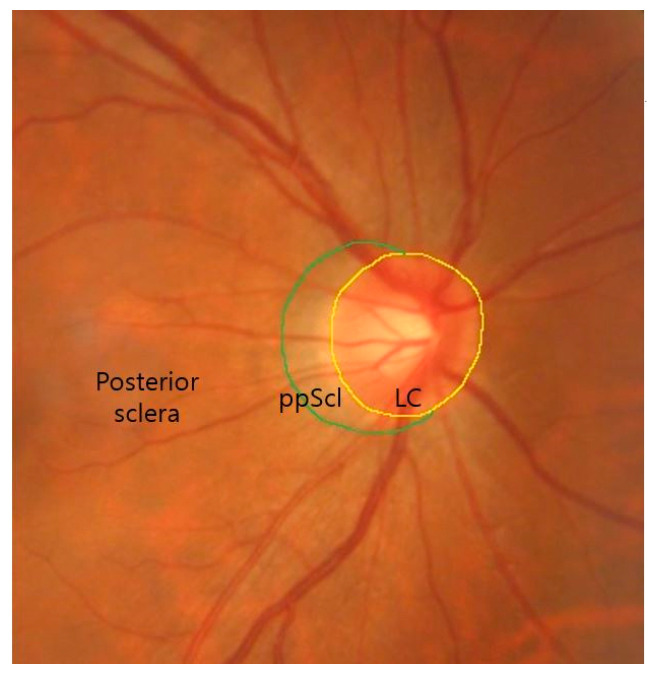
Schematic delineation of the three scleral regions surrounding the optic nerve head in a fundus image. The lamina cribrosa (LC) is outlined in yellow, the peripapillary sclera (ppScl) in green, and the adjacent posterior sclera is indicated.

Primary fibroblasts were isolated from human donor eyes obtained from an approved eye bank. Tissues from the posterior sclera, ppScl and LC were dissected under a surgical microscope (Figure 1). Small explants (approximately 1 × 1 mm) were seeded in 6-well plates coated with collagen type I (Col1a1, collagen type I alpha-1 chain; hereafter referred to as Col1) and cultured at 37 °C in a humidified incubator with 5% CO_2_. Culture medium was left for a 1 week and then was exchanged for 2–3 days using Dulbecco’s Modified Eagle Medium (DMEM) supplemented with 10% fetal bovine serum (FBS), 1% penicillin-streptomycin, and 1% sodium pyruvate. After fibroblasts migrated from the explants, cells were expanded and used at passage 5 for experiments. The study protocol was approved by the Institutional Review Board (IRB) of the Catholic University of Korea (Seoul, Republic of Korea) (KC22SISI0647), and the study design followed the tenets of the Declaration of Helsinki.

For the isolation of LC cells, enzymatic reaction method was used. LC tissues were placed in 0.3% collagenase type 2 in serum free media and left at 37 °C shaking incubator for 2 h. Cells collected after centrifuge were seeded in a Col1-coated 6-well plate and were cultured until passage 5 for experiments.

### 2.2. In Vitro Stimulation of Fibroblasts

For fibrosis and inflammation induction, fibroblasts were stimulated with TGF-β2 (5 ng/mL; Abcam, Cambridge, MA, USA), or were subjected to mechanical pressure (static pressure at ~30 mmHg for 24 h using pressure chamber). The pressure chamber used in this study was an in-house-built system, similar to the most commonly used pressurized chamber setups described in previous studies [18]. For AngII stimulation, fibroblasts were treated with AngII for 24 h. An initial concentration–response experiment was performed using 0.1, 0.3, 0.5, 0.8, and 1 µM AngII. Based on these results, where α-SMA expression was most robustly induced at 0.1 µM, the concentrations of 0.1 µM and 1 µM were selected for subsequent analyses. In separate pressure-stimulation assays, cells were treated with the TGF-β2 inhibitor Trabedersen (AP12009, 10 µM; MedChemExpress, Monmouth Junction, NJ, USA) and the AngII inhibitor SC-3062 (10 µM; Santa Cruz Biotechnology, Inc., Dallas, TX, USA) 1 h before pressure application.

### 2.3. Animal Model of Experimental Glaucoma

Adult male Sprague–Dawley rats (7–8 weeks old, weighing 250–300 g) were used in this study. All procedures adhered to the Association for Research in Vision and Ophthalmology (ARVO) Statement for the Use of Animals in Ophthalmic and Vision Research and the National Institutes of Health Guide for the Care and Use of Laboratory Animals (NIH publication No. 80-23, revised 1996). Animal care and experimental protocols were approved by the Ethics Committee and the Institutional Animal Care and Use Committee (IACUC) of the Catholic University of Korea. A total of 36 animals were included. All efforts were made to minimize the number of animals used and to ensure their welfare throughout the study.

Subtenon injections (0.1 mL) of phosphate-buffered saline (PBS), a TGF-β2 inhibitor (10 µM), or an AngII inhibitor (10 µM) were administered under inhalational anesthesia. Following injection, chronic ocular hypertension was induced by cauterization of two to three episcleral veins per eye. IOP was measured weekly using a rebound tonometer (iCare TonoLab, ICare Finland Oy, Vantaa, Finland), and animals were monitored for up to 8 weeks after cauterization.

### 2.4. Western Blot Analysis

Fibroblast lysates and scleral tissues were homogenized in RIPA buffer containing protease and phosphatase inhibitors. Protein concentrations were quantified using a BCA assay. Proteins were separated by SDS-PAGE and transferred to PVDF membranes. Primary antibodies against alpha smooth muscle actin (α-SMA), glial fibrillary acidic protein (GFAP), Col1, fibronectin, cyclooxygenase-2 (COX-2), tumor necrosis factor alpha (TNF-α), and GAPDH (all from Abcam; dilution 1:100) were used. Bands were visualized with enhanced chemiluminescence system (Amersham, Woburn, MA, USA) and were quantified using ImageMaster VDS (Pharmacia Biotech, City of Industry, CA, USA). The fold-changes in protein levels were calculated relative to GAPDH.

### 2.5. Quantitative Real-Time PCR

Quantitative real-time PCR was conducted in a 25 µL reaction mixture containing 2 µL of first-strand cDNA, 0.4 µM each of forward and reverse primers (Macrogen, Inc., Seoul, Republic of Korea), and iQ SYBR Green Supermix (Bio-Rad Laboratories, Hercules, CA, USA). Primers targeting Col1, α-SMA, fibronectin, interleukin-1β (IL-1β), interleukin-6 (IL-6), TNF-α, and COX-2 were used. The primers sequences are as follows: Col1 (Forward, GGCTACTTCTCGCTCTGCTTCATC; Reverse, TGGGCAAACTGCACAACATTCTCC), αSMA (Forward, CTATGCCTCTGGACGCACAACT; Reverse, CAGATCCAGACGCATGATG-GCA), fibronectin (Forward, GCTCTCTCTCAGACAACCATC; Reverse, CAGAGTCGCACTGGTAGAAG), IL-1β (Forward, CCACAGACCTTCCAGGAGAATG; Reverse, GTGCAGTTCAGT-GATCGTACAGG), IL-6 (Forward, AGACAGCCACTCACCTCTTCAG; Reverse, TTCT-GCCAGTGCCTCTTTGCTG), TNF-α (Forward, GGACCAGCCAGGAGGGAGAAC; Reverse, CGCCACGAGCAGGAATGAGAAG), and GAPDH (Forward, AGCTCACTGGCATGGCCTTC; Reverse, ACGCCTGCTTCACCACCTTC). Amplification was performed using the CFX96 Real-Time PCR System (Bio-Rad Laboratories), and specificity was confirmed by melt curve analysis and sequencing. For quantification, serially diluted standard curves generated from plasmid-cloned cDNA were used. mRNA expression levels were normalized to the housekeeping gene GAPDH. For each experimental group, RNA from three samples was pooled.

### 2.6. Immunocytochemistry

Fibroblasts were seeded onto coverslips coated with 0.1% gelatin in 24-well plates. Cells were fixed with 4% paraformaldehyde in PBS for 15 min at room temperature and were washed twice with PBS. Cells were then permeabilized with 0.1% Triton X-100 in PBS for 10 min and were blocked with 5% bovine serum albumin (BSA; Sigma-Aldrich Corp., St. Louis, MO, USA) in PBS for 1 h at room temperature.

Primary antibodies against α-SMA, Col1, and COX-2 (all from Abcam; dilution 1:100) were diluted in 1% BSA/PBS and were incubated overnight at 4 °C. After washing, cells were incubated with Alexa Fluor 488- or Alexa Fluor 546-conjugated secondary antibodies (Molecular Probes, Eugene, OR, USA) for 1 h at room temperature. Nuclei were counterstained with DAPI. All images were acquired using a confocal fluorescence microscope (Leica SP8; Leica Microsystems, Berlin, Germany).

### 2.7. Immunofluorescence

Enucleated rat eyes were fixed in 4% paraformaldehyde, embedded in Optimal Cutting Temperature (OCT) compound (Sakura Finetek, Torrance, CA, USA), and sectioned at 6 µm thickness. Sections of scleral and retinal tissues were stained using antibodies against α-SMA, Col1, COX-2, GFAP, and NeuN (neuronal nuclei marker). Secondary antibody binding was detected using Alexa Fluor 488- or Alexa Fluor 546-conjugated secondary antibodies (Molecular Probes, Eugene, OR, USA). Sections were mounted with anti-fade mounting medium containing DAPI. Imaging was performed using confocal laser scanning microscopy (Carl Zeiss Microscopy GmbH, Jena, Germany). Immunofluorescence intensity was quantified using ImageJ software (version 2). For each eye, three non-overlapping sections were analyzed to ensure reproducibility.

### 2.8. Retinal Ganglion Cell (RGC) Labeling

Cross-sectioned retinas were immunostained with anti-NeuN to label RGC bodies in the ganglion cell layer. Three random fields per retinal quadrant (middle region) were analyzed under 200× magnification. RGC density was quantified as the number of NeuN-positive cells per unit area.

### 2.9. Statistical Analysis

All data are presented as mean ± standard deviation (SD). Comparisons between groups were made using two-sided Student’s *t*-tests or one-way ANOVA where appropriate. A *p*-value of less than 0.05 was considered statistically significant. All experiments were performed with at least three biological replicates unless otherwise specified.

## 3. Results

### 3.1. Characterization of Human Scleral Fibroblasts and Lamina Cribrosa Cells

**Figure 2:**
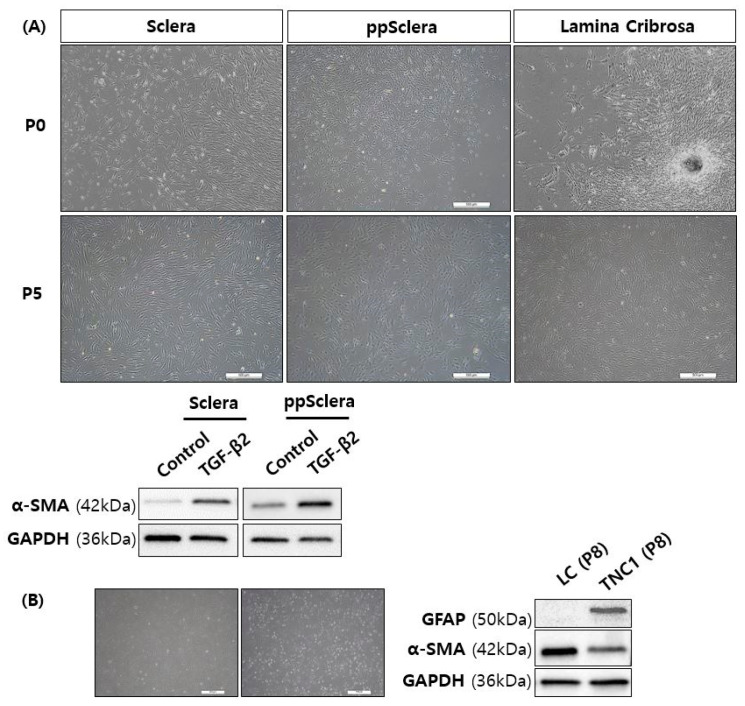
Characterization of fibroblasts from human scleral regions. (**A**) Primary fibroblasts isolated from the posterior sclera, peripapillary sclera (ppScl) and fibroblast-like lamina cribrosa (LC) cells of human donor eyes, subcultured to passage 5 (P5), and stimulated with transforming growth factor-β2 (TGF-β2). Expression of α-smooth muscle actin (α-SMA) shown in PBS- and TGF-β2–treated cells. (**B**) Fibroblasts derived from the LC stained for α-SMA and glial fibrillary acidic protein (GFAP), compared with the TNC1 astrocyte cell line. Scale bar = 500 µm. n = 3 independent experiments.

Primary fibroblasts isolated from the posterior sclera, ppScl, and fibroblast-like LC cells from the LC were successfully cultured and characterized. Fibroblasts from the sclera and ppScl exhibited increased α-SMA expression following TGF-β2 stimulation compared to PBS-treated controls in the Western blot analysis. Since LC region has both LC cells and astrocytes, we performed Western blot analysis of both α-SMA and GFAP, astrocyte marker, to confirm successful isolation of LC cells comparing with TNC1 cells (immortalized astrocyte cell line). LC cells expressed α-SMA but lacked GFAP expression, indicating a fibroblastic rather than astrocytic phenotype, whereas the TNC1 astrocytic cells expressed both GFAP and α-SMA (Figure 2).

### 3.2. TGF-β2 and Pressure Induce Fibrotic and Inflammatory Protein Expression in Fibroblasts

**Figure 3:**
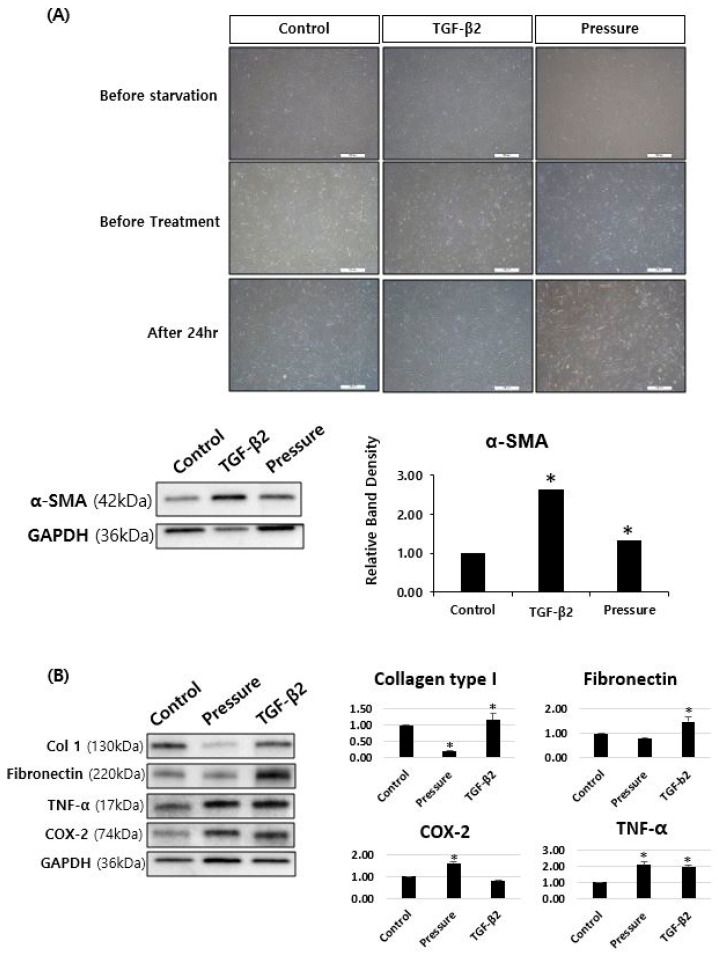
Western blot analysis of fibrotic and inflammatory protein expression in fibroblasts following stimulation with TGF-β2 and pressure. (**A**) Expression of α-smooth muscle actin (α-SMA) in fibroblasts treated with PBS, TGF-β2, or pressure. (**B**) Expression of collagen type I (Col1), fibronectin, cyclooxygenase-2 (COX-2), and tumor necrosis factor-alpha (TNF-α) under the same conditions. Scale bar = 500 µm. * Data are presented as mean ± standard deviation (SD). *p* < 0.05 compared to control. n = 3 independent experiments.

Stimulation with TGF-β2 significantly increased the expression of fibrotic markers including α-SMA, Col1, and fibronectin in fibroblasts, as shown by Western blot analysis (Figure 3). Additionally, pressure stimulation elevated inflammatory proteins such as COX-2 and TNF-α.

**Figure 4:**
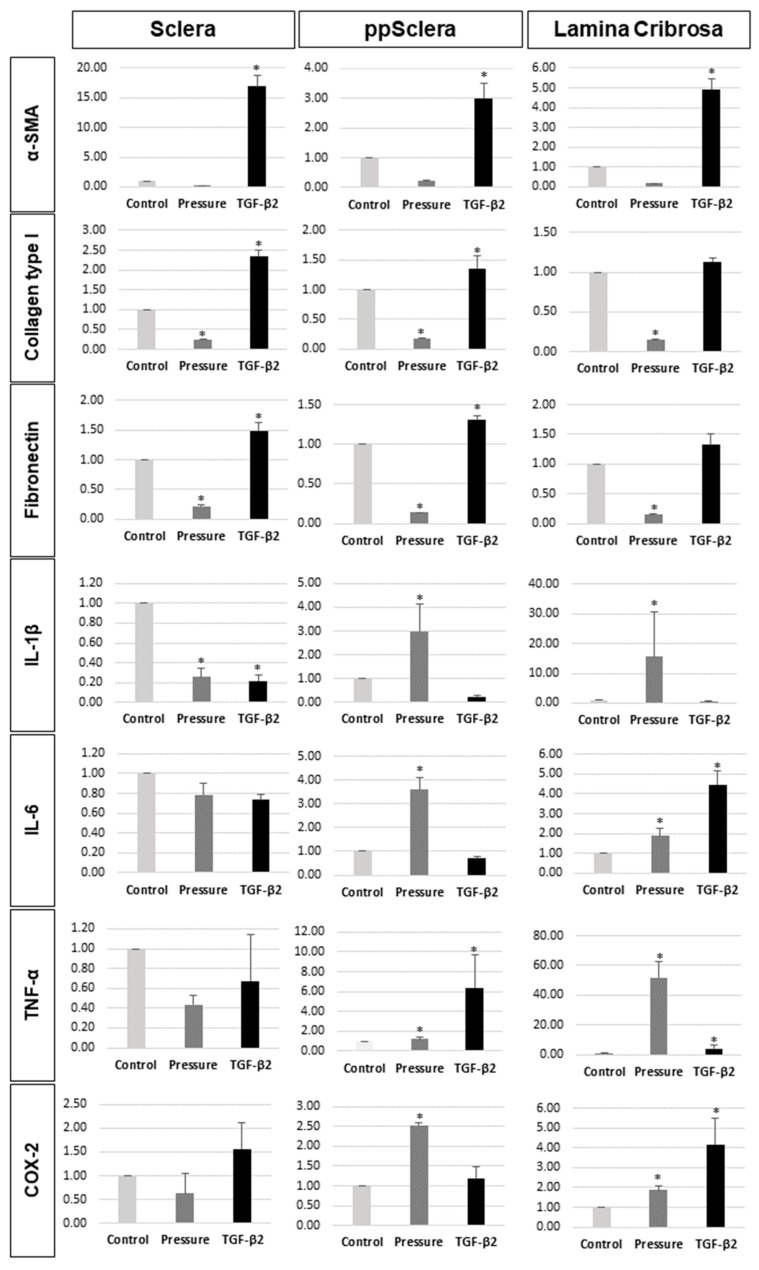
Quantitative real-time polymerase chain reaction (PCR) analysis of fibrotic and inflammatory gene expression in fibroblasts isolated from the posterior sclera, peripapillary sclera (ppScl), and fibroblast-like lamina cribrosa (LC) cells following stimulation with pressure or TGF-β2. Target genes include α-smooth muscle actin (α-SMA), collagen type I (Col1), fibronectin, interleukin-1β (IL-1β), interleukin-6 (IL-6), tumor necrosis factor-alpha (TNF-α), and cyclooxygenase-2 (COX-2). * Data are presented as mean ± standard deviation (SD). *p* < 0.05 compared to control. n = 3 independent experiments.

Quantitative real-time PCR analysis further confirmed these findings. TGF-β2 stimulation significantly increased α-SMA, Col1, and fibronectin gene expression across all three fibroblast types, whereas pressure stimulation led to a decrease in these markers relative to controls (Figure 4). Regarding inflammatory cytokines, pressure stimulation significantly upregulated IL-1β, IL-6, TNF-α, and COX-2 expression in ppScl- and LC-derived fibroblasts, but not in the posterior sclera-derived fibroblasts. TGF-β2 also increased IL-6, TNF-α, and COX-2 expression only in the LC fibroblasts (Figure 4).

**Figure 5:**
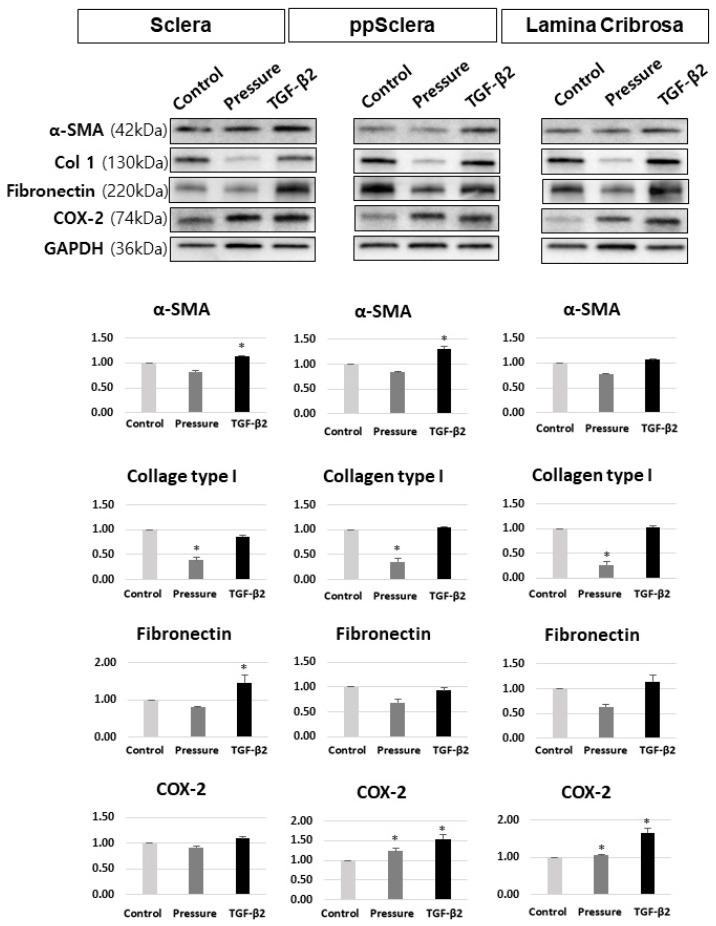
Western blot analysis of fibrotic and inflammatory protein expression in fibroblasts isolated from the posterior sclera, peripapillary sclera (ppScl), and fibroblast-like lamina cribrosa (LC) cells following stimulation with TGF-β2 and pressure. Target proteins include α-smooth muscle actin (α-SMA), collagen type I (Col1), fibronectin, and cyclooxygenase-2 (COX-2). * Data are presented as mean ± standard deviation (SD). *p* < 0.05 compared to control. n = 3 independent experiments.

Western blot analysis confirmed that TGF-β2 significantly increased the expression of α-SMA and fibronectin in fibroblasts derived from the posterior sclera. In fibroblasts from the ppScl and LC, both TGF-β2 and pressure stimulation significantly elevated the expression of COX-2 (Figure 5).

### 3.3. Angiotensin II Stimulates Fibrotic and Inflammatory Marker Expression

**Figure 6:**
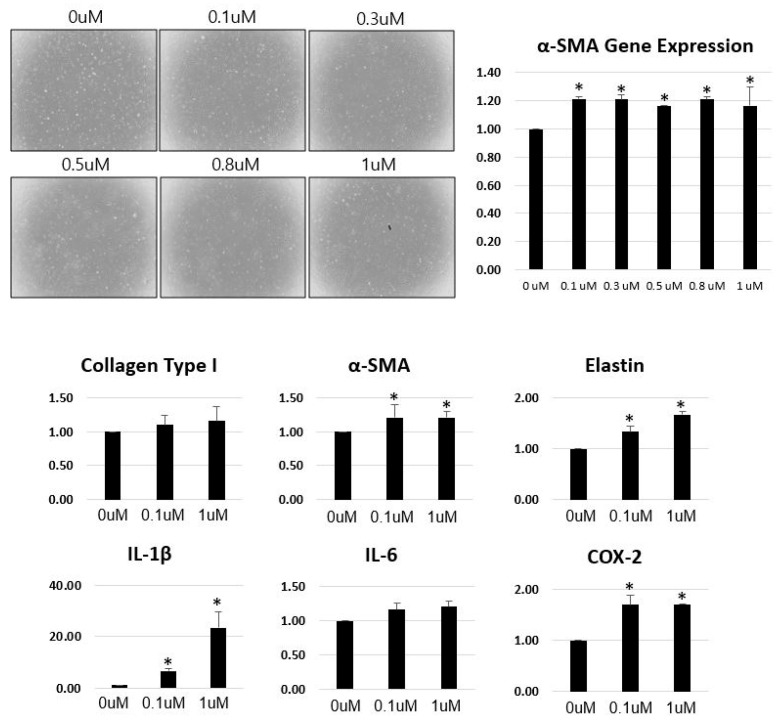
Quantitative real-time polymerase chain reaction (PCR) analysis of fibrotic and inflammatory gene expression in fibroblasts exposed to different concentrations of angiotensin II (AngII). Expression of collagen type I (Col1), α-smooth muscle actin (α-SMA), elastin, interleukin-1β (IL-1β), interleukin-6 (IL-6), and cyclooxygenase-2 (COX-2) in fibroblasts treated with 0.1 µM and 1 µM AngII. * Data are presented as mean ± standard deviation (SD). *p* < 0.05 compared to control. n = 3 independent experiments.

Treatment with AngII induced fibrotic and inflammatory changes in scleral fibroblasts. PCR analysis showed that stimulation with 0.1 µM AngII significantly upregulated α-SMA expression, leading to the selection of 0.1 µM and 1 µM concentrations for further experiments. Both α-SMA, elastin, IL-1β and COX-2 expression were significantly increased following AngII stimulation in fibroblasts (Figure 6).

**Figure 7:**
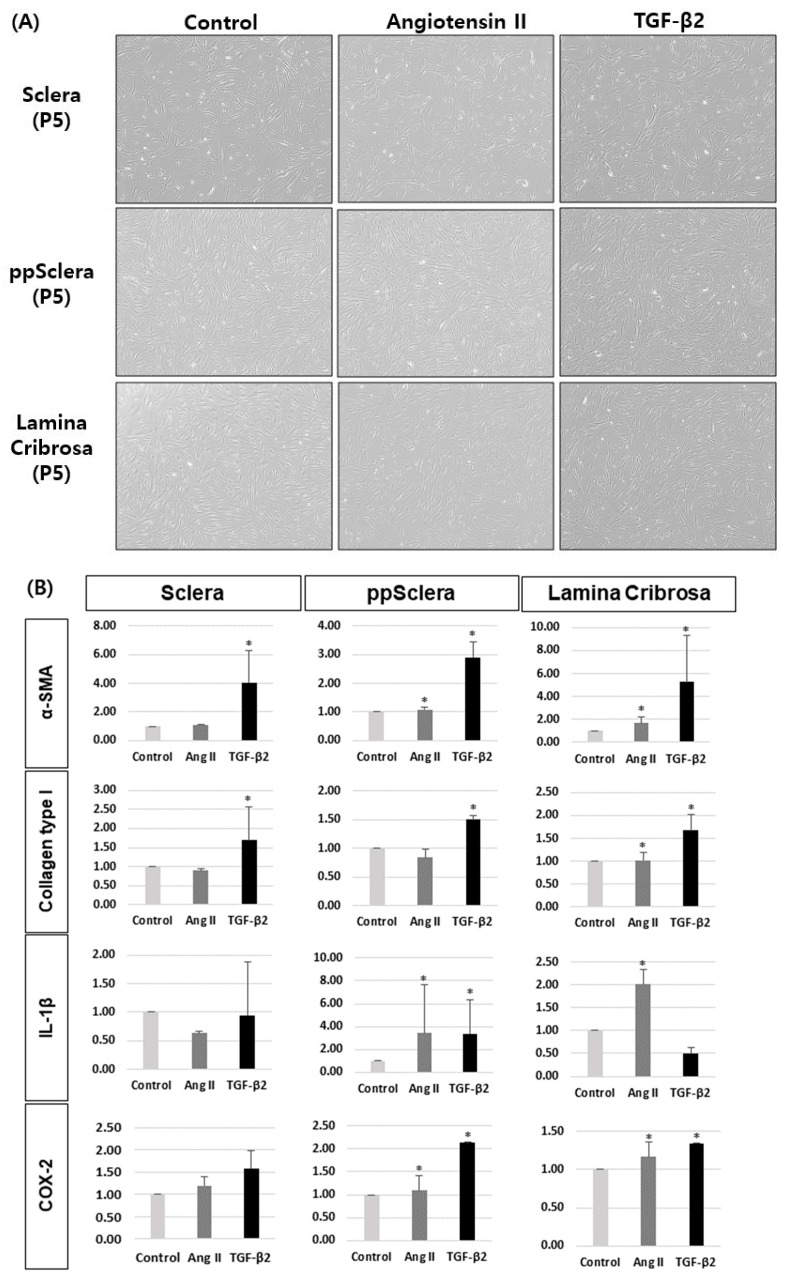
Effects of angiotensin II (AngII) and TGF-β2 on fibrotic and inflammatory gene expression in fibroblasts isolated from the posterior sclera, peripapillary sclera (ppScl), and fibroblast-like lamina cribrosa (LC) cells. (**A**) Primary fibroblasts isolated from the posterior sclera, ppScl, and fibroblast-like LC cells were subcultured to passage 5 (P5) and treated with Ang II or TGF-β2. (**B**) Quantitative PCR analysis of α-smooth muscle actin (α-SMA), collagen type I (Col1), interleukin-1β (IL-1β), and cyclooxygenase-2 (COX-2) expression in fibroblasts from each region. Data are presented as mean ± standard deviation (SD). * *p* < 0.05 compared to control. n = 3 independent experiments.

Additionally, upon treatment with AngII or TGF-β2, TGF-β2 stimulation significantly increased α-SMA and Col1 expression in fibroblstsfrom all regions, whereas AngII increased IL-1β and COX-2 expression only in ppScl fibroblasts and fibroblast-like LC cells (Figure 7).

### 3.4. Inhibitory Effects of TGF-β2 and Angiotensin II Inhibitors on Pressure-Induced Fibrotic Responses

**Figure 8:**
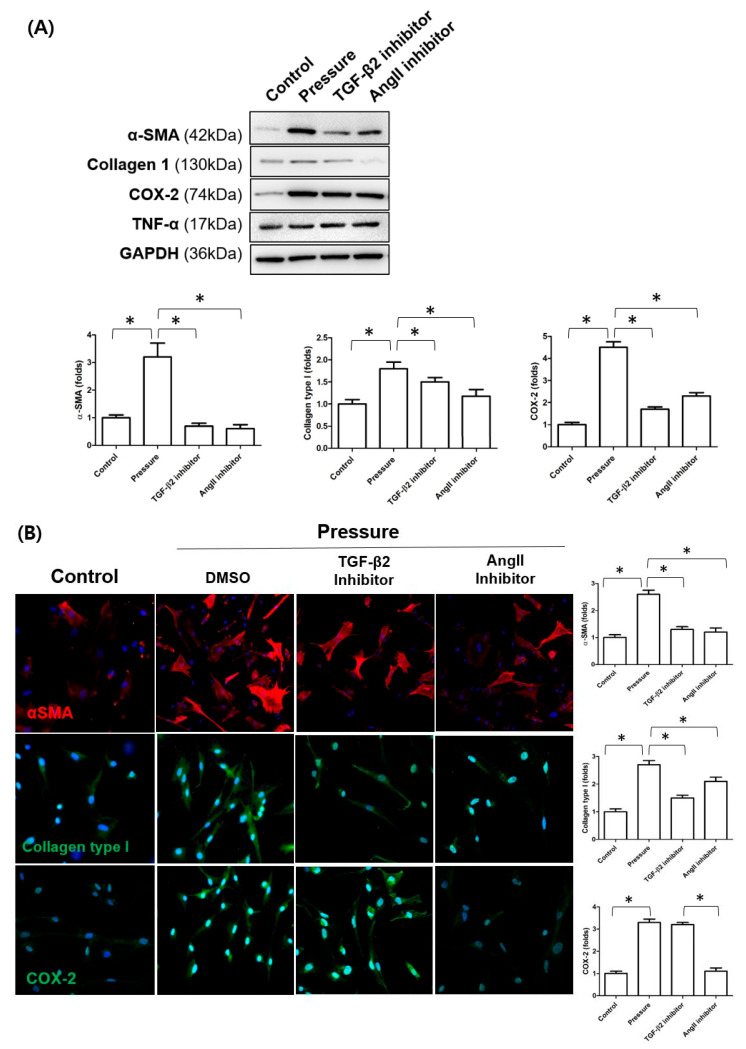
Effects of TGF-β2 and angiotensin II (Ang II) pathway inhibition on pressure-induced fibrotic and inflammatory marker expression in fibroblasts. (**A**) Western blot analysis of α-smooth muscle actin (α-SMA), collagen type I (Col1), and cyclooxygenase-2 (COX-2) expression in fibroblasts exposed to static pressure, with or without TGF-β2 or AngII inhibitor treatment. (**B**) Immunocytochemical staining of α-SMA, Col1, and COX-2 expression in fibroblasts under the same conditions. Data are presented as mean ± standard deviation (SD). * *p* < 0.05 compared to control. n = 3 independent experiments.

Pressure stimulation significantly elevated α-SMA, Col1, and COX-2 protein levels in fibroblasts. Co-treatment with either a TGF-β2 inhibitor or an AngII inhibitor significantly reduced the expression of these proteins compared to pressure stimulation alone, as shown by Western blot analysis (Figure 8A). Immunocytochemical staining confirmed the suppression of α-SMA and Col1 expression following inhibitor treatments. However, reduction in COX-2 expression was only significant after AngII inhibition in the immunocytochemical staining (Figure 8B).

### 3.5. In Vivo Effects of TGF-β2 and Angiotensin II Inhibitors in a Glaucoma Animal Model

**Figure 9:**
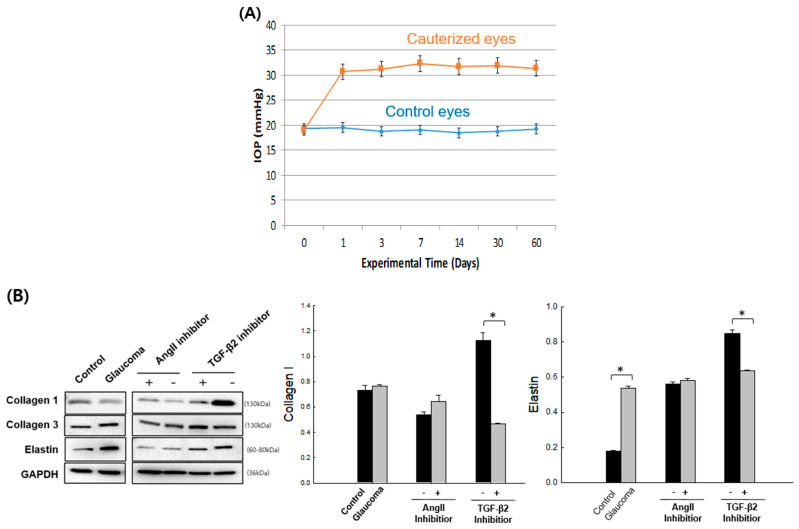

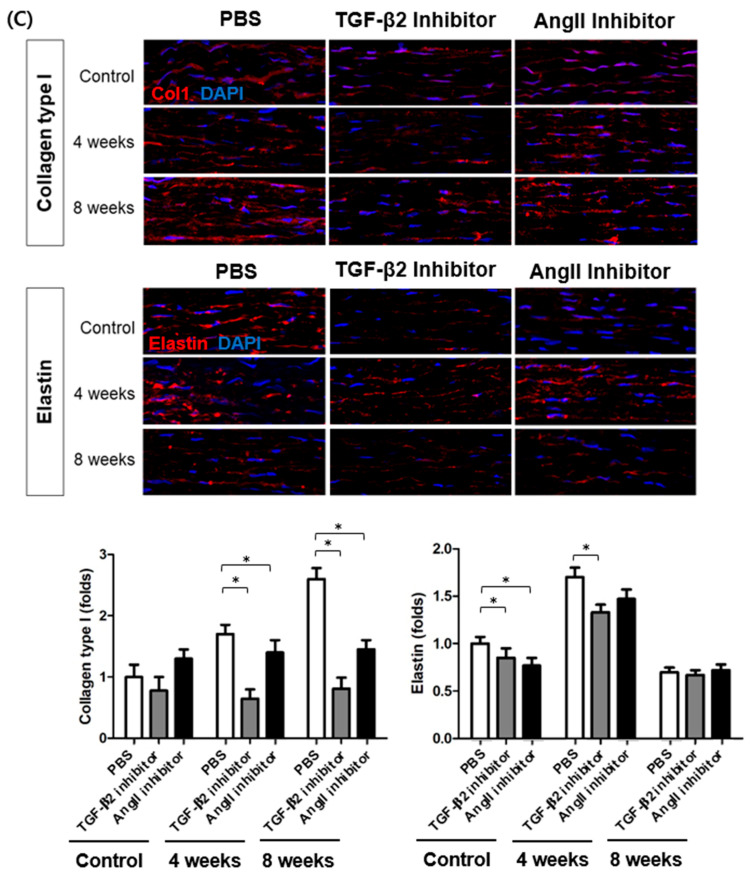
Effects of TGF-β2 and Ang II pathway inhibition in an in vivo glaucoma model. (**A**) Intraocular pressure (IOP) measurements in control and experimental eyes during the study period after subtenon injection of PBS, TGF-β2 inhibitor, or AngII inhibitor, followed by episcleral vein cauterization. (**B**) Western blot analysis of collagen type I (Col1), collagen type III (Col3), and elastin expression in control and glaucomatous eyes at 2 weeks after subtenon injection of PBS, TGF-β2 inhibitor, or AngII inhibitor. (**C**) Immunofluorescence staining of Col1 and elastin expression in scleral tissues at 4 and 8 weeks after subtenon injection of PBS, TGF-β2 inhibitor, or AngII inhibitor. * n = 6 eyes per group; total n = 36 eyes across all groups. Data are presented as mean ± standard deviation (SD). *p* < 0.05 compared to control.

In an experimental glaucoma model, a total of 36 animals were used (18 cauterized and 18 controls). Episcleral vein cauterization elevated IOP, which remained increased throughout the experimental period (Figure 9A). IOP was measured in all groups, including those treated with TGF-β2 or AngII inhibitors, and subtenon injection of either inhibitor did not significantly alter IOP compared with PBS-treated glaucomatous eyes.

Immunofluorescence analysis at 4 and 8 weeks confirmed that both TGF-β2 and AngII inhibitors decreased Col1 expression in scleral tissues, while elastin expression was reduced only by TGF-β2 inhibition at 4 weeks (Figure 9C).

### 3.6. Effects of TGF-β2 and Angiotensin II Inhibitors on Retinal Glial Activation and Neuronal Survival

**Figure 10:**
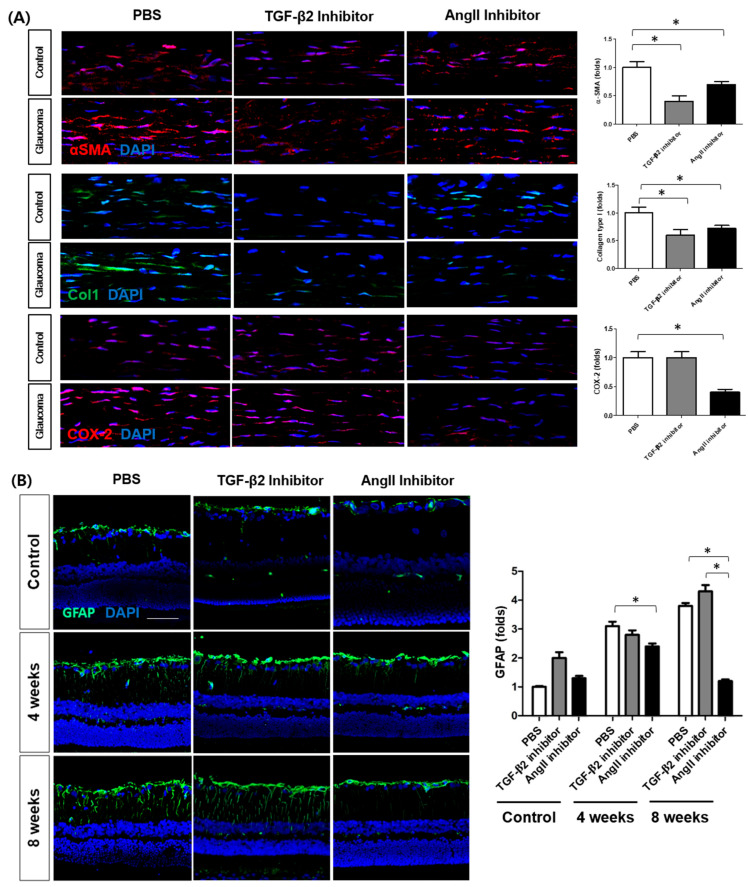

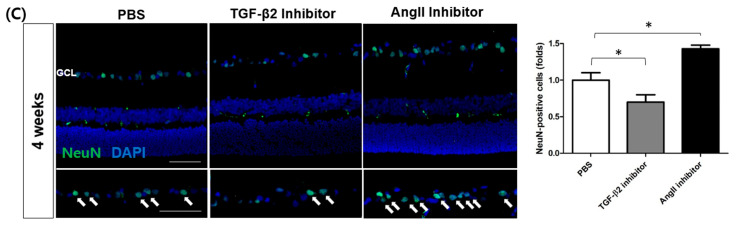
Effects of TGF-β2 and Ang II pathway inhibition on fibrotic and neuronal markers in ocular tissues from the glaucoma model. (**A**) Immunofluorescence staining of α-smooth muscle actin (α-SMA) and collagen type I (Col1) in scleral tissues from control and glaucoma (ocular hypertension, OHT) eyes. (**B**) Immunofluorescence staining of glial fibrillary acidic protein (GFAP) in retinal tissues at 4 and 8 weeks after treatment with PBS, TGF-β2 inhibitor, or AngII inhibitor. (**C**) Immunofluorescence staining of NeuN in the retinal ganglion cell layer at 4 weeks after treatment. White arrows indicate RGCs. GCL = ganglion cell layer; Scale bar = 50 µm. * n = 6 eyes per group. Data are presented as mean ± standard deviation (SD). *p* < 0.05 compared to control.

Immunofluorescence staining of the scleral tissues showed that inducing glaucoma upregulates both α-SMA and Col1 proteins. This further demonstrated that both TGF-β2 and AngII inhibitors decreased the expression of α-SMA and Col1 in glaucomatous eyes; however, the reduction was dominated by TGF-β2 inhibition. COX-2 was decreased only with AngII inhibitor (Figure 10A). In the retina, GFAP expression was markedly increased at 4 and 8 weeks after cauterization. Treatment with a TGF-β2 inhibitor slightly increased GFAP expression at 8 weeks after cauterization; however, it did not show statistical significance. Treatment with an AngII inhibitor significantly reduced GFAP expression at 4 weeks and 8 weeks (Figure 10B).

NeuN immunostaining revealed the distribution of RGC bodies in the ganglion cell layer. While elevated IOP reduced the number of NeuN-positive RGCs, TGF-β2 inhibitor further reduced the number of NeuN-positive RGCs compared to PBS-injected controls. This may suggest modulation of ECM by TGF-β2 inhibition potentiates RGC loss after elevated IOP. Ang II inhibitor significantly increased RGC survival compared to controls, suggesting that modulation of the Ang II pathway may confer both anti-inflammatory and neuroprotective effects (Figure 10C).

## 4. Discussion

This study provided further evidence that both TGF-β2 and AngII were key mediators of fibrotic and inflammatory responses in scleral fibroblasts, and contribute to the progression of glaucomatous damage through region-specific ECM remodeling. We demonstrated that fibroblasts derived from the ppScl and fibroblast-like LC cells are more responsive to TGF-β2 and AngII stimulation compared to those from the posterior sclera, particularly in terms of fibrotic marker upregulation and pro-inflammatory cytokine expression. These results supported the hypothesis that region-dependent fibroblast reactivity may contribute to differential vulnerability of ONH tissues under glaucomatous stress.

In cultured fibroblasts, TGF-β2 stimulation induced robust ECM remodeling across all scleral regions, as shown by the upregulation of α-SMA, Col1, and fibronectin. In vivo, subtenon injection of either a TGF-β2 inhibitor or an AngII inhibitor reduced ECM deposition in the sclera, with decreased expression of elastin, Col1, and α-SMA. AngII inhibition also reduced COX-2 expression, indicating that while TGF-β2 primarily promotes fibrotic changes, AngII contributes to both fibrosis and inflammation.

Pressure stimulation significantly elevated α-SMA, Col1, and COX-2 protein levels in fibroblasts, as confirmed by Western blot and immunocytochemical staining. Consistently, immunofluorescence analysis of scleral tissues in the glaucoma model showed a marked upregulation of α-SMA and Col1 following IOP elevation, highlighting the pressure-induced activation of fibrotic remodeling. This ECM remodeling likely reflects a compensatory response to mechanical stress, aimed at reinforcing the structural stiffness of the sclera and peripapillary supportive tissues. Although this response was not clearly evident in the in vitro gene expression profiles, the protein-level changes observed in both Western blot and immunocytochemistry suggest a robust and biologically relevant adaptation.

Inhibition of TGF-β2 effectively suppressed this remodeling response; however, this may interfere with the physiological stiffening required to counteract elevated IOP, potentially leading to increased tissue vulnerability and axonal damage. Given that these fibrotic responses were observed across all fibroblast regions, TGF-β2 inhibition could impair the global biomechanical resilience of the optic nerve head. In contrast, AngII exerted a less potent fibrotic effect but strongly induced pro-inflammatory cytokines in ppScl fibroblasts and fibroblast-like LC cells, implicating its role in neuroinflammation-mediated axonal injury. Suppression of this pathway improved RGC survival, suggesting that targeting AngII may provide neuroprotective benefits by mitigating inflammation without disrupting essential fibrotic adaptation.

Our in vivo results revealed that TGF-β2 inhibition primarily suppressed fibrotic remodeling without significantly reducing inflammatory responses, and failed to prevent RGC loss in the chronic ocular hypertension model. In contrast, AngII inhibition—which modulates both fibrosis and neuroinflammation—resulted in increased survival of NeuN-positive RGCs. This neuroprotective effect aligns with our previous findings, where blockade of AngII signaling via Angiotensin II receptor type I (AT1R) inhibition under systemic hypotension also led to RGC preservation [17]. These results highlight a noticeable distinction: TGF-β2 plays a central role in fibrosis, while AngII signaling contributes to mainly neuroinflammation. The absence of neuroprotection with TGF-β2 inhibition may reflect inadequate structural support due to reduced ECM deposition, leaving the peripapillary sclera vulnerable to mechanical strain. In contrast, the protective effect of AngII inhibition appears to be mediated by its suppression of neuroinflammation, which contributes to the preservation of RGCs. These findings highlight the interplay between ECM remodeling and neuroinflammatory processes in glaucoma pathogenesis and suggest that targeting the sclera may offer a novel therapeutic strategy that complements conventional IOP-lowering treatments.

Our prior publications also reported that AngII contributes to RGC loss via glial activation and necroptosis, particularly under conditions of systemic hypotension [19]. The current study supports this pathway by identifying AngII-induced expression of pro-inflammatory mediators such as COX-2, IL-1β, and TNF-α in scleral fibroblasts, further implicating the sclera as an active participant in glaucomatous neuroinflammation. The interplay between mechanical stress, fibrotic signaling, and inflammation in the ONH region appears to form a vicious cycle, whereby biomechanical deformation promotes fibroblast activation, which in turn stiffens the tissue and enhances vulnerability to further IOP-induced damage. The LC, composed of a thin, multilayered reticular connective tissue that supports the passage of RGC axons and blood vessels, represents both a region of high strain and a structural weak point [20]. Notably, our findings showed that AngII-mediated inflammatory responses were most pronounced in fibroblasts-like LC cells, as demonstrated in Figure 7, suggesting a direct contribution of AngII to axonal damage through localized inflammatory activation in this region. This observation is consistent with previous reports showing that AngII receptor upregulation in the sclera promotes fibroblast activation and ECM remodeling, particularly under systemic hypotension, ultimately leading to increased scleral stiffness and heightened vulnerability of RGCs [17].

ECM remodeling in response to elevated IOP may initially represent a protective adaptation aimed at reinforcing structural integrity of ONH. Previous studies have highlighted the regulatory roles of TGF-β2 and bone morphogenetic protein(BMP)-4 in maintaining the homeostatic balance of ECM synthesis and degradation within ONH [21]. The balancing role of BMP-4 in counteracting TGF-β2-mediated fibrotic remodeling is further supported by the protective effect of BMP-4 on RGCs and axonal regeneration, as demonstrated by enhanced RGC survival in an experimental glaucoma mouse model—suggesting that BMP-4’s function extends beyond ECM regulation to include direct support of neuronal integrity [22,23,24]. Further investigations will be needed to clarify its regulatory and neuroprotective properties in ONH remodeling.

Our study demonstrated that inhibition of ECM remodeling, particularly through TGF-β2 blockade, resulted in a paradoxical reduction in RGC survival in a chronic ocular hypertension model. While TGF-β2 inhibition effectively suppressed the expression of fibrotic markers such as α-SMA and Col1, it failed to preserve RGCs, as evidenced by decreased NeuN-positive cells. In contrast, Ang II inhibition not only attenuated fibrosis and neuroinflammation but also improved RGC survival. These observations suggest that ECM remodeling may initially play a compensatory and protective role in maintaining ONH structural integrity under glaucomatous stress. Disruption of this adaptation, particularly through unopposed inhibition of remodeling pathways such as TGF-β2, may compromise neuronal survival. Thus, the context-dependent balance between fibrotic remodeling and neuroprotection must be carefully considered in the development of targeted therapies.

The LC is structurally supported by the ppScl, a region that may experience the highest strain in response to elevated IOP [25,26,27,28]. This biomechanical coupling forms the foundation of regional vulnerability within the ONH, as highlighted by Burgoyne et al. [12], who proposed that strain-induced deformation of the LC and ppScl contributes significantly to axonal damage in glaucoma. Under chronic IOP elevation, the mechanocellular responses in these regions modify their mechanical properties, particularly stiffness, which in turn shifts local strain patterns and promotes a feedforward cycle of progressive ECM remodeling and vulnerability to injury [29,30,31]. In our study, region-specific remodeling dynamics were identified in vitro, where TGF-β-induced fibrotic changes were evident across all ONH regions, but AngII-associated neuroinflammatory responses were more pronounced in ppScl fibroblasts and fibroblast-like LC cells. These findings support the hypothesis that mechanical and inflammatory insults act synergistically in a region-dependent manner. However, since rat scleral tissues were not examined region by region, in vivo confirmation of these mechanisms remains necessary. Moreover, downstream signaling pathways such as SMAD2/3 phosphorylation, which are central to TGF-β-mediated fibrosis [8,13], were not examined. Future studies addressing both in vivo validation and signaling pathway interrogation will be important to fully elucidate the molecular mechanisms underlying scleral remodeling in glaucoma.

## 5. Conclusions

In summary, our study demonstrates that TGF-β2 and AngII distinctly contribute to glaucomatous remodeling within the ONH sclera, with TGF-β2 broadly mediating ECM deposition and AngII preferentially promoting neuroinflammatory responses, particularly within the LC and ppScl. These region-specific responses are likely influenced by the underlying biomechanical environment, which governs strain distribution and tissue susceptibility under elevated IOP. Importantly, while inhibition of either pathway attenuated fibrotic remodeling, only AngII blockade conferred significant neuroprotection, highlighting the pathological role of inflammation in glaucomatous progression. These findings underscore the necessity of therapeutic strategies that target both fibrotic and inflammatory mechanisms, tailored to the distinct regional vulnerabilities of ONH tissues. A detailed understanding of this interplay may support the development of more effective, region-targeted interventions to preserve optic nerve integrity in glaucoma.

## Data Availability

Data are available from the corresponding author upon reasonable request.

## References

[1] Resnikoff S., Pascolini D., Etya’Ale D., Kocur I., Pararajasegaram R., Pokharel G.P., Mariotti S.P. (2004). Global data on visual impairment in the year 2002. Bull. World Health Organ..

[2] Agapova O.A., Kaufman P.L., Lucarelli M.J., Gabelt B.A.T., Hernandez M. (2003). Differential expression of matrix metalloproteinases in monkey eyes with experimental glaucoma or optic nerve transection. Brain Res..

[3] Weinreb R.N., Aung T., Medeiros F.A. (2014). The pathophysiology and treatment of glaucoma: A review. Jama.

[4] Flammer J., Orgül S., Costa V.P., Orzalesi N., Krieglstein G.K., Serra L.M., Renard J.-P., Stefánsson E. (2002). The impact of ocular blood flow in glaucoma. Prog. Retin. Eye Res..

[5] Hernandez M.R., Andrzejewska W.M., Neufeld A.H. (1990). Changes in the extracellular matrix of the human optic nerve head in primary open-angle glaucoma. Am. J. Ophthalmol..

[6] Soto I., Howell G.R. (2014). The complex role of neuroinflammation in glaucoma. Cold Spring Harb. Perspect. Med..

[7] Jayaram H., Kolko M., Friedman D.S., Gazzard G. (2023). Glaucoma: Now and beyond. Lancet.

[8] Prendes M.A., Harris A., Wirostko B.M., Gerber A.L., Siesky B. (2013). The role of transforming growth factor β in glaucoma and the therapeutic implications. Br. J. Ophthalmol..

[9] Connor T., Roberts A.B., Sporn M., Danielpour D., Dart L.L., Michels R.G., de Bustros S., Enger C., Kato H., Lansing M. (1989). Correlation of fibrosis and transforming growth factor-beta type 2 levels in the eye. J. Clin. Investig..

[10] Border W.A., Noble N.A. (1994). Transforming growth factor β in tissue fibrosis. N. Engl. J. Med..

[11] Girard M.J., Suh J.-K.F., Bottlang M., Burgoyne C.F., Downs J.C. (2009). Scleral biomechanics in the aging monkey eye. Investig. Ophthalmol. Vis. Sci..

[12] Burgoyne C.F., Downs J.C., Bellezza A.J., Suh J.-K.F., Hart R.T. (2005). The optic nerve head as a biomechanical structure: A new paradigm for understanding the role of IOP-related stress and strain in the pathophysiology of glaucomatous optic nerve head damage. Prog. Retin. Eye Res..

[13] Fuchshofer R., Tamm E.R. (2012). The role of TGF-β in the pathogenesis of primary open-angle glaucoma. Cell Tissue Res..

[14] Catt K., Zimmet P., Cain M., Cran E., Best J., Coghlan J. (1971). Angiotensin II blood-levels in human hypertension. Lancet.

[15] Danser A., Derkx F., Admiraal P., Deinum J., De Jong P., Schalekamp M. (1994). Angiotensin levels in the eye. Investig. Ophthalmol. Vis. Sci..

[16] White A.J., Cheruvu S.C., Sarris M., Liyanage S.S., Lumbers E., Chui J., Wakefield D., McCluskey P.J. (2015). Expression of classical components of the renin-angiotensin system in the human eye. J. Renin-Angiotensin-Aldosterone Syst..

[17] Oh S.-E., Kim J.-H., Shin H.-J., Kim S.-A., Park C.-K., Park H.-Y.L. (2023). Angiotensin II-Related Activation of Scleral Fibroblasts and Their Role on Retinal Ganglion Cell Death in Glaucoma. Pharmaceuticals.

[18] Aires I.D., Ambrósio A.F., Santiago A.R. (2017). Modeling human glaucoma: Lessons from the in vitro models. Ophthalmic Res..

[19] Jeon S.J., Huh J., Jeong E., Park C.K., Park H.Y.L. (2022). Angiotensin II related glial cell activation and necroptosis of retinal ganglion cells after systemic hypotension in glaucoma. Cell Death Dis..

[20] Strickland R.G., Garner M.A., Gross A.K., Girkin C.A. (2022). Remodeling of the lamina cribrosa: Mechanisms and potential therapeutic approaches for glaucoma. Int. J. Mol. Sci..

[21] Zode G.S., Clark A.F., Wordinger R.J. (2009). Bone morphogenetic protein 4 inhibits TGF-β2 stimulation of extracellular matrix proteins in optic nerve head cells: Role of gremlin in ECM modulation. Glia.

[22] Ueki Y., Reh T.A. (2012). Activation of BMP-Smad1/5/8 signaling promotes survival of retinal ganglion cells after damage in vivo. PLoS ONE.

[23] Thompson A., Berry M., Logan A., Ahmed Z. (2019). Activation of the BMP4/Smad1 pathway promotes retinal ganglion cell survival and axon regeneration. Investig. Ophthalmol. Vis. Sci..

[24] Liu D., Deng Q., Lei X., Lu W., Zhao Q., Shen Y. (2021). Overexpression of BMP4 protects retinal ganglion cells in a mouse model of experimental glaucoma. Exp. Eye Res..

[25] Sigal I.A. (2009). Interactions between geometry and mechanical properties on the optic nerve head. Investig. Ophthalmol. Vis. Sci..

[26] Coudrillier B., Tian J., Alexander S., Myers K.M., Quigley H.A., Nguyen T.D. (2012). Biomechanics of the human posterior sclera: Age-and glaucoma-related changes measured using inflation testing. Investig. Ophthalmol. Vis. Sci..

[27] Downs J.C., Girkin C.A. (2017). Lamina cribrosa in glaucoma. Curr. Opin. Ophthalmol..

[28] Safa B.N., Wong C.A., Ha J., Ethier C.R. (2022). Glaucoma and biomechanics. Curr. Opin. Ophthalmol..

[29] Drance S., Anderson D.R., Schulzer M., Group C.N.-T.G.S. (2001). Risk factors for progression of visual field abnormalities in normal-tension glaucoma. Am. J. Ophthalmol..

[30] Leske M.C., Heijl A., Hussein M., Bengtsson B., Hyman L., Komaroff E., Group E.M.G.T. (2003). Factors for glaucoma progression and the effect of treatment: The early manifest glaucoma trial. Arch. Ophthalmol..

[31] Musch D.C., Gillespie B.W., Lichter P.R., Niziol L.M., Janz N.K., Investigators C.S. (2009). Visual field progression in the Collaborative Initial Glaucoma Treatment Study: The impact of treatment and other baseline factors. Ophthalmology.

